# Highlight selection of radiochemistry and radiopharmacy developments by editorial board

**DOI:** 10.1186/s41181-021-00146-9

**Published:** 2021-09-08

**Authors:** Francisco Alves, Inês F. Antunes, Emiliano Cazzola, Frederik Cleeren, Bart Cornelissen, Antonia Denkova, Jonathan Engle, Alain Faivre-Chauvet, Nic Gillings, Jeroen J. M. A. Hendrikx, Amir R. Jalilian, Nicholas P. van der Meulen, Renata Mikolajczak, Oliver C. Neels, Maroor R. A. Pillai, Raymond Reilly, Sietske Rubow, Yann Seimbille, Sarah Spreckelmeyer, Wiktor Szymanski, Carlotta Taddei

**Affiliations:** 1 University of Coimbra, Coimbra, Portugal; 2 UMCG, Groningen, The Netherlands; 3 Sacro Cuore Hospital, Verona, Italy; 4 Katholieke Universiteit, Leuven, Belgium; 5 Technische Universiteit, Delft, The Netherlands; 6 University of Wisconsin, Madison, WI USA; 7 Inserm, CNRS, Nantes, France; 8 Rigshospitalet, Copenhagen, Denmark; 9 NKI, Amsterdam, The Netherlands; 10 IAEA, Vienna, Austria; 11 PSI, Villigen, Switzerland; 12 Polatom, Otwock, Poland; 13 Helmholtz-Zentrum Dresden-Rossendorf (HZDR), Dresden, Germany; 14 Molecular Group of Companies, Cochin, Kerala India; 15 University of Toronto, Toronto, Canada; 16 Stellenbosch University, Stellenbosch, South Africa; 17 Erasmus MC, Rotterdam, The Netherlands; 18 Charite, Berlin, Germany; 19 University of Groningen, Groningen, The Netherlands; 20 NIH, Bethesda, USA

**Keywords:** Radiopharmacy, Radiochemistry, Highlight selection

## Abstract

**Background:**

The Editorial Board of EJNMMI Radiopharmacy and Chemistry releases a biyearly highlight commentary to update the readership on trends in the field of radiopharmaceutical development.

**Results:**

This commentary of highlights has resulted in 21 different topics selected by each member of the Editorial Board addressing a variety of aspects ranging from novel radiochemistry to first in man application of novel radiopharmaceuticals. Also the first contribution in relation to MRI-agents is included.

**Conclusions:**

Trends in (radio)chemistry and radiopharmacy are highlighted demonstrating the progress in the research field being the scope of EJNMMI Radiopharmacy and Chemistry.

## Introduction

Each individual member of the Editorial Board has selected a highlight article that has appeared in the radiochemistry, radiopharmacy and imaging agent literature during the period January-June 2021. The aim of this collaborative initiative is to create a biyearly overview for the readers summarizing the latest trends in the field.

### Smart MRI contrast agents enable 3D temperature mapping with high spatial resolution

#### By Wiktor Szymanski

Through an impressive application of the light-driven coordination-induced spin state switching effect to MR Imaging, an MRI contrast agent was created that enables the precise measurement of temperature (Wellm et al. 2021). This smart Ni^II^ porphyrin complex, derived from previous successful design from the same group (Venkataramani et al. 2011) features a molecular photoswitch (of azoimidazole type) that can be isomerised with light to the thermally unstable form that exhibits MRI activity, being paramagnetic. This unstable form spontaneously switches back to the stable form, which is MRI silent, in a first order process whose rate depends on the temperature. This ingenious dynamic design transfers the measurement of temperature to the time domain, relying on measuring the change of MRI signal in time, independent on its concentration and other local effects.

**Fig. 1:**
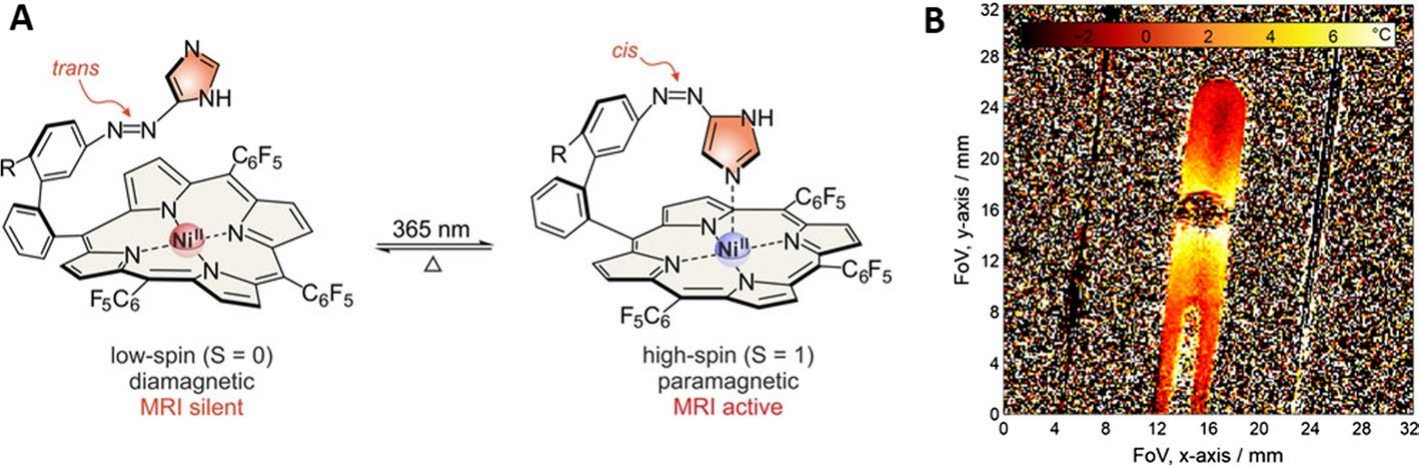
Photoswitchable MRI CA; **A** The thermally stable *trans* isomer can be switched to the metastable *cis* isomer, which will return to the initial form in a first order thermal process (on the timescale of seconds-minutes, depending on the temperature). **B** Temperature map in an NMR tube; polyethyleneimine silica beads coated with the NIR dye indocyanine green (ICG) that were warmed by infrared irradiation to act as a temperature source. (Reproduced with permission from ref. Wellm et al. (2021) under the terms of the Creative Commons Attribution-Non-Commercial License)

Temperature maps of phantoms and with local heat sources were obtained with high spatial resolutions (130 × 130 μm) and low temperature errors (< 0.22 °C) (Fig. 1). The limitations of the current system include the low water solubility (measurements were performed in methanol) and overall low relaxivity of the CA (estimated as ~ 0.3 s^− 1^ mM^− 1^). However, as a proof-of-principle, this study presents a ground-breaking new approach to MRI temperature measurements and further developments towards clinical applications are eagerly awaited.

### Guideline on current good radiopharmacy practice (cGRPP) for the small-scale preparation of radiopharmaceuticals

#### By Renata Mikolajczak

Radiopharmaceuticals are considered a special group of medicines. Therefore, their preparation and use are regulated by a number of European Union directives, regulations and rules that member states have adopted. In addition, this regulatory landscape is changing continuously. How to meet that challenge in a way covering all the aspects related to the small-scale preparation of radiopharmaceuticals at nonindustrial sites (hospital pharmacies, nuclear medicine departments, PET centres)—this question is addressed in the article entitled: “Guideline on current Good Radiopharmacy practice (cGRPP) for the small-scale preparation of radiopharmaceuticals” representing the view of the Radiopharmacy Committee of the European Association of Nuclear Medicine (EANM) (Gillings et al. 2021).

Next to the documents on Good Radiopharmacy Practices such as (1) PIC/S guide to good practices for the preparation of medicinal products in healthcare establishments, (2) GMP Annex 3 describing radiopharmaceuticals, (3) European Pharmacopeia monograph 5.19. extemporaneous preparation of radiopharmaceuticals, and (4) other specific EANM guidelines, this guideline document is aimed to assist radiopharmacies in the small-scale preparation of radiopharmaceuticals in a way safe for human use, whether from licensed or not-licensed starting materials.

In the light of positive experience with the first EANM Guidelines on cGRRP in the preparation of Radiopharmaceuticals published in 2007, the new guideline is welcomed by radiopharmacists as a great aid in their everyday routines.

### Radioimmunotherapy much more promising than immunotherapy in pancreatic cancer treatment

#### By Antonia Denkova

Pancreatic cancer remains one of the deadliest cancer types with life expectancy of just 4–6 months, if the disease has metastasized. In such cases chemotherapy and immunotherapy can be applied, but both show rather limited improvement of patient outcome. However, radioimmunotherapy might offer hope for patients having pancreatic cancer. In a recent paper (Aghevlian et al. 2020) radioimmunotherapy was compared to immunotherapy. The monoclonal antibody panitumumab can be used to target the over-expressed epithermal growth factor receptors on pancreatic cancer cells. In this study panitumumab was radiolabeled with both ^111^In (an Auger emitter) and ^177^Lu (a β^−^ emitter) and evaluated in vitro and in vivo. This particular monoclonal antibody appears to have endogenous nuclear translocation sequence which facilitates uptake by the cell nucleus, explaining the choice of ^111^In. The in vitro results showed that the ^177^Lu-labeled antibody was much more efficient than the ^111^In-variant which was attributed to the cross-fire effect of the beta minus emitter (i.e. multiple cells are hit in the surrounding of the targeted cell). In vivo, both radiolabeled antibodies showed decrease in tumour growth, while the monoclonal antibody alone did not have any effect on tumour growth retardation. Also here ^177^Lu appeared to be more effective, requiring much less activity to achieve the same effect when compared to ^111^In. However, in both cases no side effects were observed which allows the draw the conclusion that radioimmunotherapy has a potential of treating pancreatic cancer. Hopefully, this paper will inspire more studies on this topic.

### Cold kits: the next big step for ^68^Ga-radiopharmaceuticals?

#### By Oliver C. Neels

Radiopharmaceuticals obtained from cold kits radiolabeled with ^99m^Tc have been the working horse in Nuclear Medicine for several decades. The same applies for [^18^F]FDG in PET radiopharmacies, with limitations in tumor entities with low glycolytic activity. ^68^Ga-labelled radiopharmaceuticals have shown to overcome these limitations with the most prominent examples targeting somatostatin receptors or the prostate-specific membrane antigen (PSMA). Recent developments show the potential of cold kits in combination with ^68^Ge/^68^Ga generators (Satpati 2021). Currently, a variety of ^68^Ge/^68^Ga generators with or without marketing authorization is available on the market complemented by the increasing popularity of cyclotron-produced ^68^Ga (Rodnick et al. 2020; Thisgaard et al. 2021). The challenges during reconstitution of cold kits are the use of different volumes of eluate and buffer for pH adjustment and different amounts of ligand, all depending on the source and formulation of ^68^Ga resulting in different methods how to produce a single batch of a radiopharmaceutical. Industry has put in efforts to establish licensed cold kits (e.g. SomaKit TOC, NETSPOT), while other cold kits addressing targets like PSMA, gastrin releasing peptide receptors (GRPR), integrin receptors or bacterial infections are available without marketing authorization. Cold kits might be an economic alternative to the current method of automated production of ^68^Ga-radiopharmaceuticals keeping current good radiopharmacy practice in mind (Gillings et al. 2021).

### Use of in silico models for optimization of ^177^Lu-DOTATATE therapy

#### By Jeroen Hendrikx

In silico modeling, like physiologically-based pharmacokinetics (PBPK) modeling, are routinely used in drug development to predict pharmacokinetic processes of drugs and to evaluate effects of age, diseases and drug-drug interactions (Rowland et al. 2011). In PBPK models, a database of physiological population parameters (like body composition, (organ) blood flow and receptor expression) is combined with drug data (like administered dose, renal clearance rate and receptor binding affinity) to simulate blood and organ concentrations of the drug over time. These models can also be used to optimize clinical study designs and select dosing strategies. For radioligand-based therapies, the use of PBPK models can help to select the best radioligand(s) for clinical efficacy in an early stage by estimation of tumor and organ uptake based on (radio)chemical characteristics and receptor affinity. An example of the use of a PBPK model to guide future clinical trials for dose optimization is recently published (Jiménez-Franco et al. 2021). In this study, minimal tumor perfusion rates and receptor density for therapy efficacy (in terms of 99% tumor control probability) of receptor radionuclide therapy (PRRT) with ^177^Lu-DOTATATE were estimated. Estimations after three different treatment strategies were virtually compared: (1) standard therapy (4 cycles of 7.4 GBq with 105 nmol (~ 150 µg) peptide); (2) 7.4 GBq/105 nmol for as many cycles as possible taking into account the maximum biological effective dose for kidney and bone marrow (BED_max_); (3) 4 cycles with a dose and peptide amount that is optimized to reach the BED_max_ in 4 cycles. Results show that the minimal tumor perfusion rate needed for therapy efficacy is lower for all treatment strategies than average tumor perfusion rates observed in patients. However, minimal tumor receptor expression levels needed for therapy efficacy are higher for standard therapy than for the other strategies. Although this cannot directly be translated to daily clinical practice, this study shows the potential to compare and select treatment strategies or ligands (when different peptides are compared) based on PBPK modeling prior to clinical trial design.

### Automatization of [^177^Lu]Lu-FAPI-derivatives—a step towards standardized radiolabeling with lutetium-177

#### By Sarah Spreckelmeyer

The fibroblast activation protein (FAP) is omnipresent these days, due to the successful application of FAP inhibitors with radionuclides in imaging and therapy of multiple malignant cancer types. Thus far, different FAP-derivatives can be radiolabeled with gallium-68 (Spreckelmeyer et al. 2020; yttrium-90 or lutetium-177 (Lindner et al. 2019), actinium-225 or copper-64 (Watabe et al. 2020). In the highlighted research paper, the automated production of [^177^Lu]Lu-FAPI-04 and [^177^Lu]Lu-FAPI-46 is described (Eryilmaz and Kilbas 2021).

The following parameters for the automated synthesis were used:

- Buffer: 50 mg ascorbic acid + 7.9 mg NaOH dissolved in 1 mL ultrapure water.
- Temperature: 95 °C.
- Reaction time: 20 min.
- Concentration of Lutetium-177: 100 mCi in 100 µL 0.04 HCl.
- Amount of FAPI-04/FAPI-46: 100 µg (preliminary studies performed with 15, 25 and 100 µg).
- Synthesizer: Modular Lab Eazy from Eckert and Ziegler.

The description of GMP-compliant quality control methods (e.g. radiochemical purity by HPLC and TLC) and the evaluation of the stability over 4 days at 24 °C and in human serum at 37 °C leads to a valuable synthesis protocol of [^177^Lu]Lu-FAPI-04 and [^177^Lu]Lu-FAPI-46.

### Is ^203^Pb/^212^Pb the next holy radionuclide theranostic pair?

#### By Yann Seimbille

Targeted radionuclide therapy is on the rise and matched theranostic radioisotope pairs, such as lead-203 and lead-212, hold great promise. By making use of TRIUMF’s particle accelerator capability, McNeil et al. have been able to produce ^212^Pb, as well as its imaging surrogate ^203^Pb (McNeil et al. 2021). Lead-212 was obtained from a ^228^Th/^212^Pb generator, while bombardment of thallium-203 targets on a 13 MeV cyclotron afforded lead-203. Thorium-208 was isolated as a by-product of the ^232^Th proton spallation used to produce ^225^Ac on TRIUMF’s main 500 MeV cyclotron. Then, a simple purification process based on solid phase extraction has been established, allowing direct radiolabeling and reduction of radiation exposure. Both ^203^Pb and ^212^Pb were obtained with high radionuclidic purity (> 99%), moderate yield (^203^Pb: 73.8%, ^212^Pb: 69.3%) and chemical purity suitable for pre-clinical applications. Labeling efficiency of both radioisotopes were evaluated with commercially available chelates (DOTA and TCMC) and a series of pyridine-based cyclen analogs (DOTA-xPy, x = 1–3). Quantitative chelation (RCYs > 95%) of Pb isotopes was observed at room temperature for all chelates at a concentration of 10^− 4^ M. However, at lower concentration, higher radiolabeling yields were observed with pyridine-based cyclen chelators. Therefore, it was postulated that DOTA-3Py may be a good candidate to complex ^203/212^Pb for theranostic studies. These encouraging results are paving the road for further development of lead-labeled theranostics and their future translation to clinic, but scale up efforts and investigation on Pb(II) coordination chemistry are still required.

### A phase I study of a PARP1-targeted topical fluorophore for the detection of oral cancer

#### By Bart Cornelissen

Genomic instability in tumours, because of, and causing, mutational load, is an important characteristic of cancer cells. In line with this, the DNA-damage repair enzyme poly-(ADP-ribose)-polymerase (PARP) is upregulated in cancer tissue, compared to normal tissue. A raft of fluorescently and radiolabelled compounds, based on PARP inhibitors that bind to the NAD^+^ binding pocket of the enzyme, have been reported in the literature in recent years (Chan et al. 2021; Carney et al. 2017). Several of these have been translated to clinical trials: ^18^F-FTT and ^18^F-PARPi. In a population of ovarian or head-and-neck cancer patients, respectively, uptake of these radiolabelled compounds in tumour tissue was demonstrated (Makvandi et al. 2018; Schoeder et al. 2020).

Similarly to those radiolabelled compounds, it was shown that fluorescently labelled versions of these PARP inhibitors could highlight PARP-expressing tumour tissue, compared to surrounding normal tissue. When exposed to the fluorescently labelled PARP inhibitor, PARP-FL, the tumour cells will take up and retain the compound, which can then be visualised using fluorescence imaging, highlighting tissue to be removed, and confirm removal of tumour margins post resection.

In the highlighted paper, Demétrio de Souza et al. showed rather elegantly, that, after gargling with a solution of PARP-FL, tumour tissue could be identified and removed in a series of oral cancer patients, despite its fluorescence in the green part of the light spectrum, generally seen as sub-optimal for optical imaging (Demetrio de Souza Franca et al. 2021).

Such paired sets of fluorescent and radiolabelled show great promise to guide diagnosis, localisation and resection of tumour tissue, based on molecular imaging of a cancer hallmark.

### Detectives in radiochemistry

#### By Sietske Rubow

In radiopharmacy, we get excited about new products and interesting synthesis methods. We should ask ourselves if we give sufficient attention to unusual factors that affect radiochemical yield, and purity of radiopharmaceuticals, as well as possible problems with approved and even pharmacopoeial analytical methods.

A recent paper describes a series of investigations after observing that both the precursor and its reference standard of [^68^Ga]Ga-PSMA-11 are unstable in aqueous solutions (Iudicello et al. 2021). The authors demonstrate that contamination with the omnipresent Fe(III) ion during radiosynthesis and during the synthesis of the precursor, can contribute to low radiolabelling yield and impurities in the radiopharmaceutical, and cause difficulties with the pharmacopoeial analytical method for radiochemical purity. They also suggest a remedy to stabilise the reference standard in solution.

The paper shows logical progression in answering questions about the stability of compounds, the nature of impurities, and possible solutions for problems. This paper can remind us, especially those of us who are not regularly involved in developing and validating new methods, of the importance of critical consideration of all steps during validation of synthesis and analytical methods.

### Reducing the salivary gland uptake of PSMA radioligands

#### By Raymond M. Reilly

PSMA-binding radioligands (e.g. ^177^Lu-PSMA-617 and ^225^Ac-PSMA-617) have shown promise for treatment of metastatic prostate cancer (Jones et al. 2020). However, a major limitation is high salivary gland uptake causing xerostomia (“dry mouth”) which decreas e patient quality-of-life (Heynickx et al. 2021). Salivary gland uptake is not solely due to PSMA binding since PSMA in the salivary glands is low and heterogeneous (Kratochwil et al. 2018). Moreover, ^177^Lu-J591 anti-PSMA monoclonal antibodies did not reveal salivary gland uptake in patients (Tagawa et al. 2019). Thus, there may be non-specific mechanisms that explain the salivary gland uptake of PSMA peptide radioligands. Felber et al. (2021) studied modifications to the glutamate domain of ^177^Lu-labeled PSMA-10 to reduce salivary gland uptake. These included (1) modifications to the zinc-binding site, (2) construction of pro-inhibitors that require PSMA hydrolytic cleavage, and (3) insertion of substituents at the P1′-γ-carboxylic acid position. Most modifications decreased PSMA binding affinity or internalization by LnCaP human prostate cancer cells in vitro. All modified ligands exhibited lower tumour uptake in vivo in CB17-SCID mice with LnCaP xenografts than ^177^Lu-PSMA-10. Salivary gland uptake was not reduced. Although these modifications proved not successful, there remains the potential to design PSMA radioligands with lower salivary gland accumulation.

### FAPI-74 PET/CT using either ^18^F-AlF or Cold-Kit ^68^Ga labeling: biodistribution, radiation dosimetry, and tumor delineation in lung cancer patients

#### By Emiliano Cazzola and Jonathan Engle

Since their explosive adoption as the radiolabel to diagnostics for prostate cancer, ^68^Ga-radiopharmaceuticals have rapidly expanded into other clinical applications recent years due to the ease of ^68^Ga incorporation into novel drugs, successful advances and dissemination of ^68^Ga production technologies, and strong medical industry support. Theranostic applications followed naturally in parallel with the exploration of novel, ß^−^emitting targeting agents, all facilitated by surprising, successful regulatory approvals for these important drugs. In multiple instances, ^68^Ga-labeled novel drugs have generated additional interest in development of fluorinated analogs. This manuscript capitalizes on the versatility of aluminum-fluoride synthons as alternatives to more traditional radiometal chelation in the context of FAPI-radiopharmaceuticals, which are already amassing interest that will precede marketing authorization requests. Here, Giesel et al. further demonstrate the relevance of ^18^F-FAPI tracers and overcome the limitations to theranostic application of ^18^F with creative use of a metallic fluorine synthon (Giesel et al. 2021).

### The drive to introduce scandium radioisotopes into a clinical setting

#### By Nicholas P. van der Meulen

Scandium radioisotopes have gained momentum towards its use in radiotheragnostics over the last decade. Scandium-44, a radionuclide suitable for PET imaging, has been produced via the elution from a titanium-44/scandium-44 generator, however, more recently, developments have occurred to allow it to be produced by means of compact medical cyclotrons. Scandium-43, also a PET nuclide, on the other hand, has been more problematic—and expensive—to produce. Interest has been shown in the therapeutic scandium radioisotope, scandium-47. Various means have been demonstrated, and discussed, to produce this desired radionuclide, however, each means discussed thus far has its shortcomings.

In the meantime, the scandium radioisotopes have been labeled to various chelators towards preclinical studies, as well as first-in-human applications as a proof of principle.

While it is clear that further development is necessary to optimize the production of scandium nuclides, another step to help smoothen its path into the clinic is discussed in this extremely detailed review (Mikolajczak et al. 2021). This paper candidly discusses the possibility of standardized procedures for scandium-based radiopharmaceuticals to pave the way towards potential European Pharmacopeia monographs for the perspective scandium radioisotopes in question. In particular, for the PET scandium radioisotopes, it can be argued that scandium-43/44 mixtures will have no impact on patient safety, as well as image quality of images since both are positron emitters with similar half-life. The potentially co-produced scandium-44 m decays to scandium-44 with low-energy gamma emission which will not harm the patient, however, a limit of scandium-44 m co-production should be established to ensure unnecessary radiation doses to the patient is avoided. These particular points will be raised in any discussion with authorities for permission to use the radiopharmaceutical and it is important to candidly discuss it whenever possible.

### Multi-curie production of gallium-68 on a biomedical cyclotron and automated radiolabelling of PSMA-11 and DOTATATE

#### By Amir R. Jalilian

**Fig. 2:**
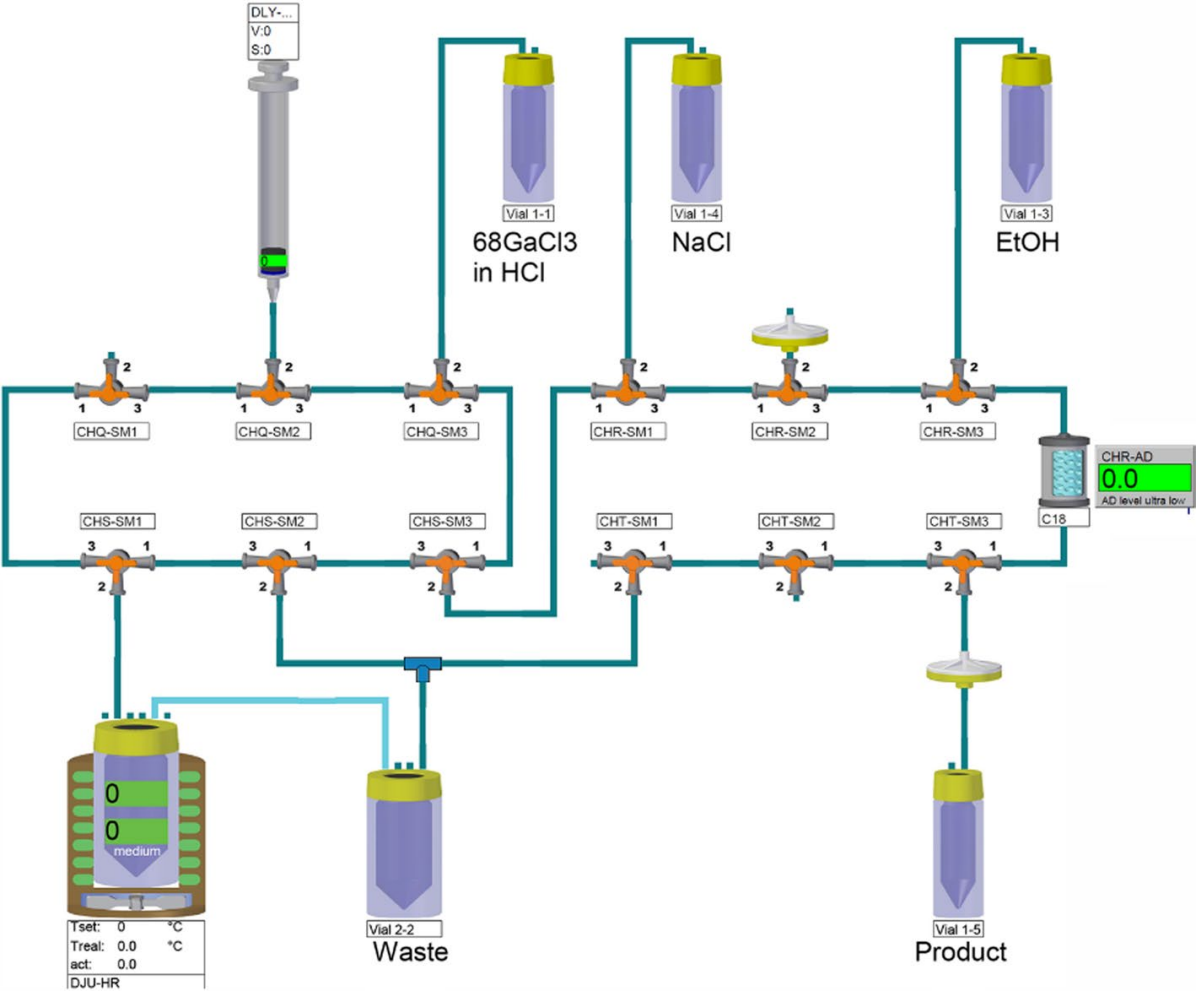
Overview of the automated synthesis cassette for radiolabelling of PSMA-11. This figure was obtained from Thisgaard et al. (2021) which was licensed under a Creative Commons License http://creativecommons.org/licences/by/4.0/

Gallium-68 has become an important role-player in theranostic radiopharmacy. Allthough many centres have been utilising generator-based [^68^Ga]GaCl_3_, high costs, long waiting lists and limited daily output of commercial generators have led to interests on ^68^Ga-cyclotron production using liquid and solid targets, supported by industries and international initiatives (IAEA 2019). Liquid targets (usually nitric acid solutions) has been widely used in various centres worldwide (Pandey et al. 2019) to produce [^68^Ga]GaCl_3_, however some concerns on the limited yields as well as acid effects on the surfaces have been reported. On solid targets, using electroplated zinc targets are widely reported in the literature. Many teams intend to apply ready to use, standard, commercially available options such as pressed powder, foils etc. (Alnahwi et al. 2019). A recent article in *EJNMMI Radiopharmacy and Chemistry* describes a direct [^68^Ga]GaCl_3_ automated production method in high yields from solid zinc-68 targets and followed clinical-grade, [^68^Ga]Ga-PSMA-11 and [^68^Ga]Ga-DOTATATE production (Thisgaard et al. 2021). Irradiated enriched metallic zinc-68 targets (up to 80 µA, 13 MeV protons, 120 min) led to the production of up to 194 GBq (5.24 Ci) of [^68^Ga]GaCl_3_ (end of purification) followed by application of a fully automated dissolution/separation process compliant to Ph. Eur. (radiochemical purity > 99.9%, radionuclidic purity sufficient for a shelf-life of up to 7 h) (Fig. 2) (Ph Eur, 2019). The report describes the production of up to 72.2 GBq [^68^Ga]Ga-PSMA-11 (> 98.2%) as well as 3.2 GBq DOTATATE (> 95%). Decision on application of generator based or cyclotron based [^68^Ga]GaCl_3_ is dependent on various factors, such as number of daily required doses, availability of cyclotron, hot cells and trained staff as well as local regulatory aspects.

### [^211^At]Astatine: an innovative radionuclide fully validated for alpha therapy clinical trials

#### By Alain Faivre-Chauvet

The place of vectorized alpha therapy is not well defined and must be evaluated through new clinical trials. After [^223^Ra]Radium dichloride, which is the only alpha emitter with a marketing authorization for an application limited to prostate bone metastases, other radionuclides are being studied to treat small tumor metastases, including: [^225^Ac]Actinium, [^213^Bi]Bismuth, [^212^Pb]Lead/[^212^Ac]Bismuth, [^211^At]Astatine, and [^227^Th]Thorium. Each radionuclide has advantages and disadvantages linked in particular to their physical and chemical properties. The publication by Lindegren et al. (2020) highlights the place of one of them: [^211^At]Astatine which is probably one of the most promising alpha emitters for clinical applications. The physical properties of [^211^At]Astatine (half life: 7.2 h and a single radioactive daughter in the disintegration chain) are described as fully suited to radiotherapy vectorized by peptides and antibodies and should allow centralized radiopharmaceutical production. However, the chemical properties of [^211^At]Astatine, in particular the low stability of the C-At bond compared to the C-I bond, represents for the authors a drawback even if the stability of astatinated radiopharmaceuticals is sufficient for clinical applications. Another advantage of [^211^At]Astatine is the relatively easy production by 30 MeV cyclotrons with the ^209^Bi(α, 2n)^211^At reaction on a solid target even though the cross section of the ^209^Bi(α, 3n)^210^At reaction starts at 28 MeV and increases with the energy beam. Even though Astatine has metallic properties, the most widely described way to produce radiopharmaceuticals with this radionuclide are electrophilic or nucleophilic substitutions on aryl groups. The most promising method to produce astatinated antibodies is the possibility to obtain this type of radiopharmaceuticals in one step. This process can lead to the development of fully automated astatine radiolabelling which should increase the availability of this type of radiopharmaceutical for clinical use.

### Combination radionuclide therapy: a new paradigm

#### By Maroor R.A. Pillai

A review article has been published on ‘Combination radionuclide therapy: A new paradigm’ which collates the results of clinical studies combining targeted radionuclide therapy with other modes of therapy such as chemotherapy, adjuvant therapy and external beam radiation therapy (EBRT) (Suman et al. 2021). They reported 31 clinical studies combining RNT agents including [^177^Lu]DOTATATE, [^177^Lu]PSMA-617, and selected ^177^Lu-labeled monoclonal antibodies in combination with different chemotherapeutic regimens. The authors have quoted the clinical response observed in the different trials, which might help in enhancing such clinical studies.

RNT in combination with molecular targeted adjuvants such as radiosensitizers provides an alternative approach wherein the DNA repair pathways are blocked by using agonists, antagonists, or inhibitors thereby achieving radio sensitization and reducing the side-effects of high-dose therapy. The authors have listed a number of ongoing and four completed Phase I/II trials, the results of which indicate a reasonable response of treatment. RNT along with EBRT is another combination therapy the authors have discussed. The authors discussed 18 clinical trials giving details of the dose, radiopharmaceutical and the efficacy of therapy. Combination radionuclide therapy is very likely to garner greater thrust in the years to come, augmenting the role of therapeutic radiopharmaceuticals in mainstream cancer management.

### Improved therapeutic index for pretargeted radioimunotherapy by using a self-assembling and disassembling platform

#### By Frederik Cleeren

Two-step pretargeted radioimmunotherapy (PRIT), where the unlabeled tumor-targeting IgG is delivered separately from the radioisotope, has improved therapeutic indices compared to conventional IgG-based radioimmunotherapy (IgG-RIT), but often still results in myelotoxicity due to unbound IgG molecules capturing and circulating the radioisotopes. Clearing agents can be used to remove excess antibodies from circulation, but the resulting three-step approach complicates translation to the clinic.

**Fig. 3:**
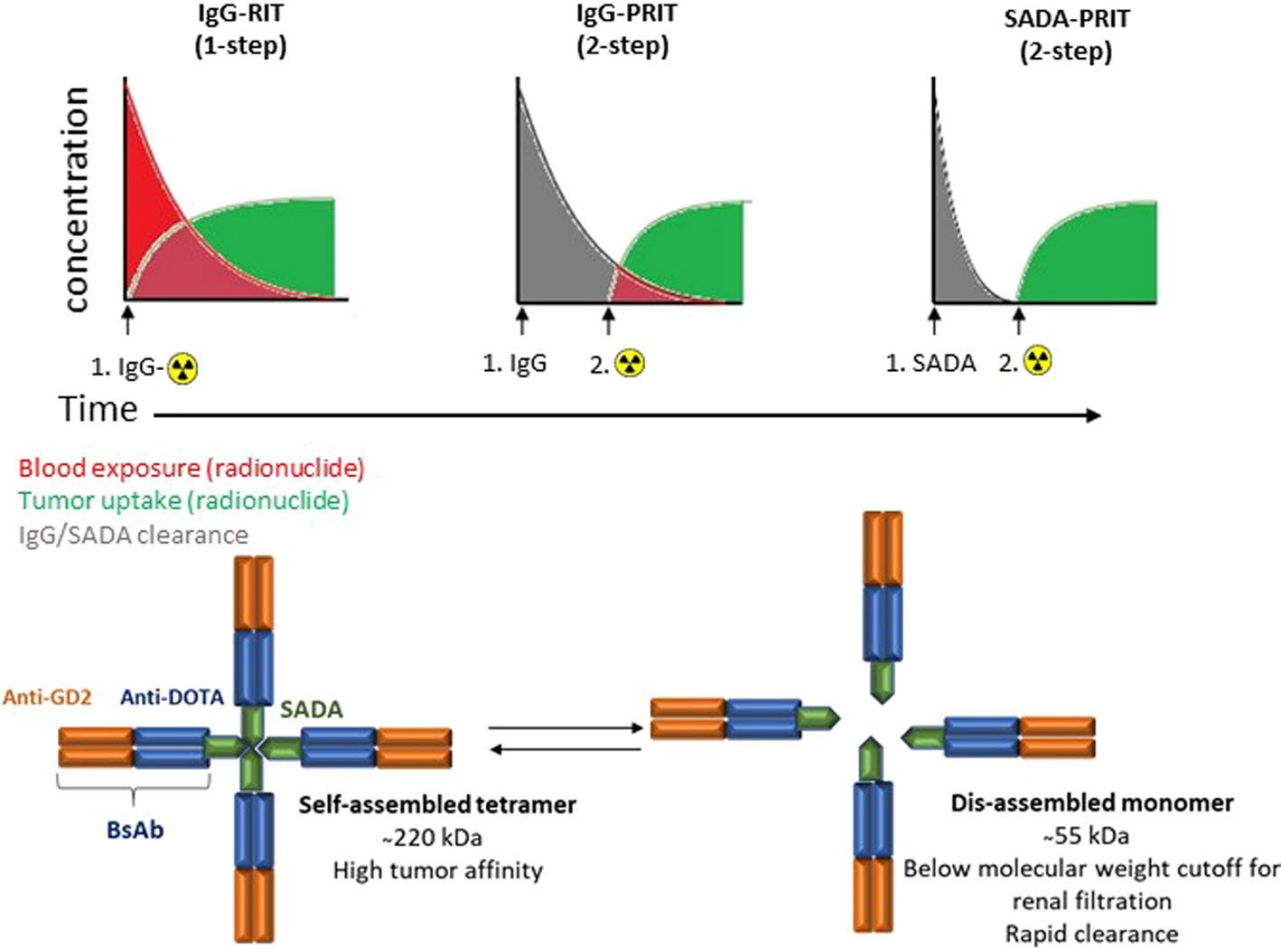
Schematic overview of convential IgG-radioimmunotherapy (IgG-RIT) and 2-step IgG-pretargeted radioimmunotherapy (IgG-PRIT), compared to self assembling and disassembling pretargeted radioimmunotherapy (SADA-PRIT). Each P53-SADA-BsAb monomer consists out of three domains: an antitumor domain (orange), an anti-DOTA domain (blue), and a SADA domain (green). Self-assembled tetramers disassemble into monomers that are rapidly cleared from circulation

To address these problems, a new class of radiopharmaceuticals, based on a self-assembling and disassembling (SADA) bispecific antibody (BsAb) platform used in a two step PRIT setting was published (Santich et al. 2021). This platform consists out of P53 tetramerized domain-based SADA domains fused to a tandem single-chain BsAb, targeting both anti-ganglioside GD2 and DOTA-caged radionuclides. SADA–BsAbs self-assemble into stable tetramers (220 kDa), but disassemble after a period of circulating in the blood (hours) into dimers or monomers (55 kDa), that are then rapidly cleared via renal filtration (Fig. 3). The GD2 specific P53-SADA-BsAbs were successfully tested in a two-step PRIT setting using ^86^Y-DOTA as the diagnostic payload, and ^177^Lu-DOTA or ^225^Ac-proteus-DOTA as the therapeutic payload, injected 48 h after injection of P53-SADA-BsAbs.

Excellent tumor-to-background PET/CT images were acquired without the need of a clearing agent. One round of the two-step treatment using 37 kBq of ^225^Ac-proteus-DOTA was sufficient to control both neuroblastoma and small-cell lung carcinoma patient derived xenografts without any clinical or histological toxicities to the bone marrow, liver, kidneys, spleen, or bone. SADA-BsAbs are a unique robust platform for effective targeted delivery of radioisotopes and because its modularity, they can be adapted to most tumor antigens.

### The use of HEPES-buffer in the production of gallium-68 radiopharmaceuticals—time to reconsider strict pharmacopeia limits?

#### By Francisco Alves

HEPES buffer is extensively used in the production of Gallium-68 compounds and numerous studies have proven its superior properties for pH control during the radiosynthesis of gallium-68 radiopharmaceuticals. This zwitterionic buffer is, nevertheless, not very common in pharmaceutical preparations where more “physiological” buffers such as phosphate, acetate or citrate are preferred and, therefore, current limits in the regulatory documentation are quite strict.

The European Pharmacopoeia prescribes a limit of 200 µg of HEPES per injected dose, what is considered a challenge considering that is not always simple to avoid it from the final preparation especially considering the tendency in the field to move from automated synthesis to ready-to-use cold kits. Furthermore, the method described in the European Pharmacopoeia to test for HEPES has proven to be unreliable and, therefore, producers struggle to comply with the current limits.

In this Letter to the Editor (le Roux et al. 2021), the authors review the available data on the toxicity of HEPES based on published data in the literature. An animal study in dogs concluded that no serious adverse effects were associated with HEPES in doses up to 500 mg/kg. In humans, HEPES was evaluated for cancer treatment with doses up to 5 g administered 2–3 times per week with minimal side effects and, recently, FDA approved a drug with an average recommended dose of 126 mg of HEPES per administration.

Additionally, the authors pointed out that better optimised methods for HEPES analysis of Ga-68 radiopharmaceuticals have been published (Pfaff et al. 2018; Antunes et al. 2020), leading to more reliable quality control tests for this substance.

The authors conclude that the regulatory institutions should review the current limits for HEPES in Gallium-68 radiopharmaceuticals as well as the current recommended methods for its determination.

### Broad scope and high-yield access to unsymmetrical acyclic [^11^C]ureas for biomedical imaging from [^11^C]carbonyl difluoride

#### By Nic Gillings

In order to benefit from the huge potential offered by carbon-11 radiopharmaceuticals for basic research and diagnostic imaging, methods which can be easily translated into routine use are required. Robust synthetic routes which avoid harsh chemicals and reaction conditions, are not overly sensitive to trace amounts of water or oxygen along with the possibility for straightforward automation are required to realise this potential.

[^11^C]Carbonyl difluoride is produced readily from cyclotron produced [^11^C]CO_2_, which is converted online to [^11^C]CO and then passed through a column containing silver difluoride at room temperature to almost quantitively yield [^11^C]carbonyl difluoride. An initial report demonstrated this synthon’s potential for the synthesis of a range of cyclic substrates such as imidazolidin-2-ones, thiazolidin-2-ones and oxazolidin-2-ones (Jakobsson et al. 2020). This was achieved by the reaction of [^11^C]carbonyl difluoride at room temperature in polar aprotic solvents such as acetonitrile using very limited amounts of precursors (down to 0.25 mg) and giving moderate to high radiochemical yields (30–96%). Furthermore, reaction times were short (7 min) and small amounts of water (1%) did not reduce radiochemical yields.

**Fig. 4:**
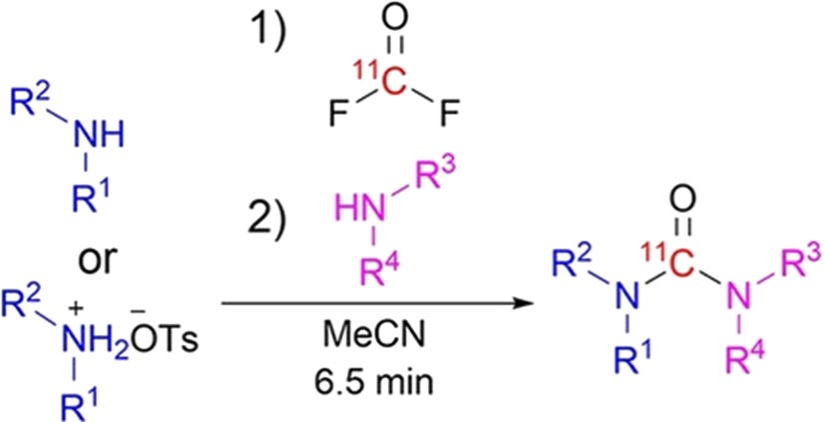
The rapid and high-yield syntheses of unsymmetrical acyclic [^11^C]ureas under mild conditions (room temperature and within 7 min) using no-carrier-added [^11^C]carbonyl difluoride with aliphatic and aryl amines are described. This new methodology is compatible with a range of functional groups and with both aliphatic amines and anilines

The latest report demonstrates that this new methodology also enables the preparation of acyclic [^11^C]ureas (Jakobsson et al. 2021) using both aliphatic amines and anilines, and is compatible with a range of functional groups (Fig. 4). Thus, using [^11^C]carbonyl difluoride as a radiolabelling synthon, there is scope for the fully automated preparation of complex PET radiotracers under mild conditions.

### Boost of trifluoromethylation agents for radiochemistry application

#### By Carlotta Taddei

The trifluoromethyl moiety (–CF_3_) is becoming increasingly prevalent in drug candidates and is currently found in many pharmaceuticals, such as fluoxetine. Due this increasing prominence, methodologies to produce ^18^F-trifluoromethylated PET radiotracers are in expansion. Reported methods implement [^18^F]fluoroform or its copper(I) derivative as ^18^F-trifluoromethylation agents to synthesize a variety of ^18^F-trifluoromethylated compounds (Yang et al. 2019). The interest in expanding the availability of ^18^F-trifluoromethylation agents motivated this highlighted article (Pees et al. 2021) in developing ^18^F-Ruppert–Prakash reagent ([^18^F]Me_3_SiCF_3_), a trifluoromethylation agent commonly used in organic synthesis. This work initially followed a non-radiochemical procedure (Prakash et al. 2012) to produce [^18^F]Me_3_SiCF_3_ from [^18^F]fluoroform reacted with trimethylsilyl chloride in toluene in the presence of potassium hexamethyldisilazide (KHMDS). Following optimization of [^18^F]Me_3_SiCF_3_ synthesis and purification by distillation over solid phase extraction (SPE) cartridge, they obtained [^18^F]Me_3_SiCF_3_ in radiochemical yields of up to 85–95% and radiochemical purities of 95% within 20 min. The versatility of [^18^F]Me_3_SiCF_3_ as ^18^F-trifluoromethylation agent was explored with substituted benzaldehydes, acetophenones and benzophenones to produce the desired ^18^F-trifluoromethylated products. Reaction variables, such as temperature, type of initiator and amount of precursor, were explored. The corresponding ^18^F-trifluoromethylated compounds were synthesized in radiochemical yields of 3–96% at room temperature within 5 min. The authors noted that the high amount of precursor (200 µmol) needed requires further optimization for routine application of PET radiotracers synthesis. Furthermore, there is scope for improving the molar activity (13 ± 2 GBq/µmol) and the overall radiochemical yield (11 ± 3%). In conclusion, this study reports the first synthesis and application of [^18^F]Me_3_SiCF_3_ to produce ^18^F-trifluoromethylated molecules. In addition, the production of [^18^F]Me_3_SiCF_3_ enables to expand the development of novel ^18^F-trifluoromethylation strategies for the design of ^18^F-trifluoromethylated PET radiotracers.

## Conclusions

Trends in radiochemistry and radiopharmacy are highlighted demonstrating the progress in the research field being the scope of EJNMMI Radiopharmacy and Chemistry.

## Data Availability

Datasets mentioned in this article can be found in the cited articles.

## Abbreviations

DNA: Deoxy ribonucleic acid
EANM: European Association of Nuclear Medicine
cGRPP: Current Good Radiopharmacy practice
EBRT: External beam radiation therapy
FAP: Fibroblast activation protein
GRPR: Gastrin-releasing peptide receptor
MRI: Magnetic resonance imaging
PARP: Poly-(ADP-ribose)-polymerase
PBPK: Physiologically-based pharmacokinetics
PRIT: Pretargeted radioimmunotherapy
PSMA: Prostate-specific membrane antigen
RCY: Radiochemical yield
RNT: Radionuclide therapy
